# *Zanthoxylum beecheyanum* enhances vancomycin activity and inhibits biofilm formation in resistant *staphylococcus aureus*

**DOI:** 10.3389/fphar.2026.1919407

**Published:** 2026-08-19

**Authors:** Arshed Idrees, Ibrahim Salih Abbas, Jian Li, Maytham Hussein, Tony Velkov

**Affiliations:** 1 Department of Pharmacognosy, College of Pharmacy, Mustansiriyah University, Baghdad, Iraq; 2 Department of Microbiology, Monash Biomedicine Discovery Institute, Monash University, Clayton, VIC, Australia; 3 Department of Pharmacology, Monash Biomedicine Discovery Institute, Monash University, Clayton, VIC, Australia

**Keywords:** adjunctive therapy, biofilm inhibition, MRSA, *Staphylococcus aureus*, vancomycin synergy, *Zanthoxylum beecheyanum*

## Abstract

The increasing prevalence of multidrug-resistant *Staphylococcus aureus* continues to compromise the effectiveness of conventional antimicrobial therapy and highlights the need for novel adjunctive treatment strategies. Despite the traditional medicinal use of *Zanthoxylum* species, the antibacterial potential of *Zanthoxylum beecheyanum* remains largely unexplored. Here, we characterized the chemical composition of *Zanthoxylum beecheyanum* essential oil (ZBEO) and evaluated its antibacterial activity against a panel of clinically relevant *S. aureus* strains, its ability to potentiate vancomycin activity, and its effects on biofilm formation. GC-MS profiling identified D-limonene and methyl cinnamate as the predominant volatile metabolites. ZBEO exhibited antibacterial activity against all tested strains, with MIC values ranging from 8 to 128 mg/L. Checkerboard assays demonstrated synergistic effects between ZBEO and vancomycin, with fractional inhibitory concentration index values as low as 0.375. Time-kill assays showed that ZBEO exhibited antibacterial activity as monotherapy and, in combination, enhanced bacterial killing and reduced bacterial regrowth compared with vancomycin alone. In addition, ZBEO inhibited biofilm formation in a concentration-dependent manner, with the greatest activity observed during combination treatment with vancomycin. Preliminary safety assessment demonstrated low hemolytic activity and limited cytotoxicity toward HEK-293 cells (CC_50_ > 128 mg/L) within the antibacterial concentration range. These findings identify ZBEO as a promising adjunct to vancomycin for enhancing antibacterial activity and inhibiting *S. aureus* biofilm formation, warranting further investigation as a plant derived adjunctive therapy for difficult-to-treat staphylococcal infections.

## Introduction

Multidrug-resistant *Staphylococcus aureus* remains a major global public health threat (Antimicrobial Resistance, 2022; Koh et al., 2024). Methicillin-resistant *S. aureus* (MRSA), in particular, is a leading cause of bloodstream, respiratory, skin and soft tissue, and device-associated infections, with limited treatment options contributing to substantial morbidity and mortality (Hussein et al., 2020; Tong et al., 2015). The emergence of glycopeptide intermediate phenotypes, including heterogeneous vancomycin-intermediate *S. aureus* (hVISA) and vancomycin-intermediate *S. aureus* (VISA), further limits the effectiveness of vancomycin therapy and complicates clinical management (Howden et al., 2010). In addition, the ability of *S. aureus* to form biofilms further contributes to persistent infection by restricting antibiotic penetration and promoting bacterial survival within a protected extracellu1lar matrix (Costerton et al., 1999; Otto, 2018).

Vancomycin remains a cornerstone therapy for serious MRSA infections; however, reduced susceptibility and persistent infections increasingly compromise its clinical efficacy (Canty et al., 2021; Cong et al., 2020). Consequently, considerable efforts have focused on identifying adjunctive agents capable of enhancing antibiotic activity, suppressing bacterial regrowth, and inhibiting biofilm formation (Worthington and Melander, 2013; Hussein et al., 2025a). Natural products and plant derived secondary metabolites continue to represent a valuable source of structurally diverse compounds with antibacterial, antibiofilm, and antibiotic potentiating properties (Newman and Cragg, 2020; Atanasov et al., 2015; Hussein et al., 2025a; Allemailem, 2021). Recent studies have demonstrated that medicinal plant extracts possess activity against MRSA and MSSA and remain an important source of novel anti-staphylococcal agents (Kulandhaivel et al., 2026). Several phytochemicals, including flavonoids, alkaloids, terpenoids, and phenolic acids, have been reported to disrupt bacterial membrane integrity, interfere with quorum sensing pathways, and inhibit biofilm development. In addition, these compounds can potentiate the activity of conventional antibiotics against resistant pathogens (Cushnie and Lamb, 2005; Afonso et al., 2023; Silva et al., 2016).

The genus *Zanthoxylum* (family Rutaceae) comprises numerous medicinal plant species traditionally used in Asian, African, and Middle Eastern ethnomedicine for the treatment of infectious and inflammatory conditions (Okagu et al., 2021a; Okagu et al., 2021b; Wen et al., 2024). Extracts and essential oils derived from *Zanthoxylum* species have demonstrated a broad range of biological activities, including antibacterial, antifungal, anti-inflammatory, and antioxidant effects. These beneficial activities are largely attributed to their chemically diverse phytochemical composition, including alkaloids, lignans, coumarins, flavonoids, and volatile terpenoids (Wen et al., 2024; Okagu et al., 2021b; Okagu et al., 2021a; Okagu et al., 2021b). Antibacterial activity has also been reported for essential oils from other *Zanthoxylum* species, including *Zanthoxylum bungeanum*, against bacterial pathogens (Zheng et al., 2024). Although species-specific ethnomedicinal information for *Zanthoxylum beecheyanum* remains limited, its close taxonomic relationship to medicinally important members of the genus, together with its largely unexplored phytochemical profile, provides a strong ethnopharmacological rationale for investigating its antimicrobial potential. Among the major volatile constituents previously identified in *Zanthoxylum* essential oils are D-limonene, methyl cinnamate, β-pinene, and caryophyllene, several of which have independently demonstrated antimicrobial and antibiofilm activities (Sun, 2007; Kotan et al., 2008; Okagu et al., 2021a).

Although antimicrobial activity has been reported for several *Zanthoxylum* species, comparatively little is known about the activity of *Z*. *beecheyanum* against clinically relevant resistant *S. aureus* phenotypes, particularly those exhibiting reduced vancomycin susceptibility and enhanced biofilm forming capacity. Furthermore, limited information is available regarding the ability of *Z. beecheyanum* essential oil to enhance vancomycin activity, suppress bacterial regrowth, or inhibit biofilm formation. In this study, we characterized the chemical composition of *Z. beecheyanum* essential oil and evaluated its antibacterial activity, potential synergy with vancomycin, and antibiofilm activity. Preliminary *in vitro* safety was also assessed using hemolysis and HEK-293 cytotoxicity assays. We show that *Z. beecheyanum essential oil* possesses intrinsic antibacterial and antibiofilm activity and enhances the activity of vancomycin against multiple resistant *S. aureus* strains, supporting its further investigation as a plant derived adjunctive therapy for difficult-to-treat staphylococcal infections.

## Results

### Essential oil extraction from fresh leaves

Fresh leaves of *Z*. *beecheyanum* K. Koch were subjected to hydrodistillation using a Clevenger apparatus for 3 h. A measured portion of fresh leaves (100.10 g) yielded approximately 1.0 mL of essential oil, corresponding to an approximate yield of ∼1% (v/w) based on fresh plant material. The essential oil was clear to pale yellow and was collected for subsequent GC-MS analysis.

### GC-MS profiling of *Zanthoxylum beecheyanum* essential oil

GC-MS analysis of the *Z*. *beecheyanum* essential oil revealed a volatile profile dominated by monoterpenes and aromatic ester metabolites. The total ion chromatogram showed two dominant peaks at retention times of 10.326 and 16.001 min, corresponding to D-limonene and methyl cinnamate, respectively (Supplementary Figure S1). These two compounds accounted for most of the detected volatile oil composition.

D-limonene was the predominant metabolite, representing 50.85% of the total peak area, followed by methyl cinnamate [(E)-3-phenyl-2-propenoic acid methyl ester] at 38.30%. Other detected volatile metabolites included β-pinene (6.22%), 1,4,7-cycloundecatriene, 1,5,9,9-tetramethyl-, Z,Z,Z- (1.56%), caryophyllene (1.16%), cyclohexene, 1-methyl-4-(1-methylethylidene)- (0.72%), α-terpinyl acetate (0.61%), and octanoic acid methyl ester (0.58%). The major volatile metabolites are summarized in Supplementary Table S1.

### Antibacterial activity of the *Zanthoxylum beecheyanum* essential oil

ZBEO demonstrated antibacterial activity against all tested *S*. *aureus* strains, with MIC values ranging from 8 to 128 mg/L (Table 1). The lowest MIC value was observed against the MSSA reference strain *S. aureus* ATCC 29213 (8 mg/L). Among resistant strains, the hVISA strain Mu3 (ATCC 700698) exhibited an MIC of 16 mg/L, whereas MRSA strains including ATCC 43300, JKD6008, and JKD6009 had MICs of 32 mg/L.

**TABLE 1:** Antibacterial activity of *Zanthoxylum beecheyanum* essential oil (ZBEO) against *Staphylococcus aureus* strains.

Strain	Phenotype	ZBEO MIC (mg/L)
*S. aureus* ATCC 29213	MSSA^a^	8
*S. aureus* ATCC 43300	MRSA^b^	32
*S. aureus* ATCC 700699 (Mu50)	VISA^c^	64
*S. aureus* ATCC 700698 (Mu3)	hVISA^d^	16
*S. aureus* JKD6008	MRSA	32
*S. aureus* JKD6009	MRSA	32
*S. aureus* 20,691	Clinical isolate (MRSA)	64
*S. aureus* 17,107	Clinical isolate (MRSA)	64
*S. aureus* 17,084	Clinical isolate (MRSA)	128
*S. aureus* 17,120	Clinical isolate (MRSA)	64
*S. aureus* 21,471	Clinical isolate (MRSA)	64
*S. aureus* 440,092	Clinical isolate	32
*S. aureus* 439,819	Clinical isolate	64
*S. aureus* 439,668	Clinical isolate	64
*S. aureus* 440,090	Clinical isolate	128

^a^ MSSA, methicillin-susceptible *Staphylococcus aureus*.

^b^ MRSA, methicillin-resistant *Staphylococcus aureus*.

^c^ VISA, vancomycin-intermediate *Staphylococcus aureus*.

^d^ hVISA, heterogeneous vancomycin-intermediate *Staphylococcus aureus*.

Higher MIC values were observed for the VISA strain Mu50 (ATCC 700699) and several clinical isolates, ranging from 64 to 128 mg/L. Overall, ZBEO retained measurable antibacterial activity against MSSA, MRSA, hVISA, VISA, and multiple clinical *S. aureus* isolates (Table 1).

### Time-kill activity of ZBEO monotherapy against *Staphylococcus aureus*

**FIGURE 1:**
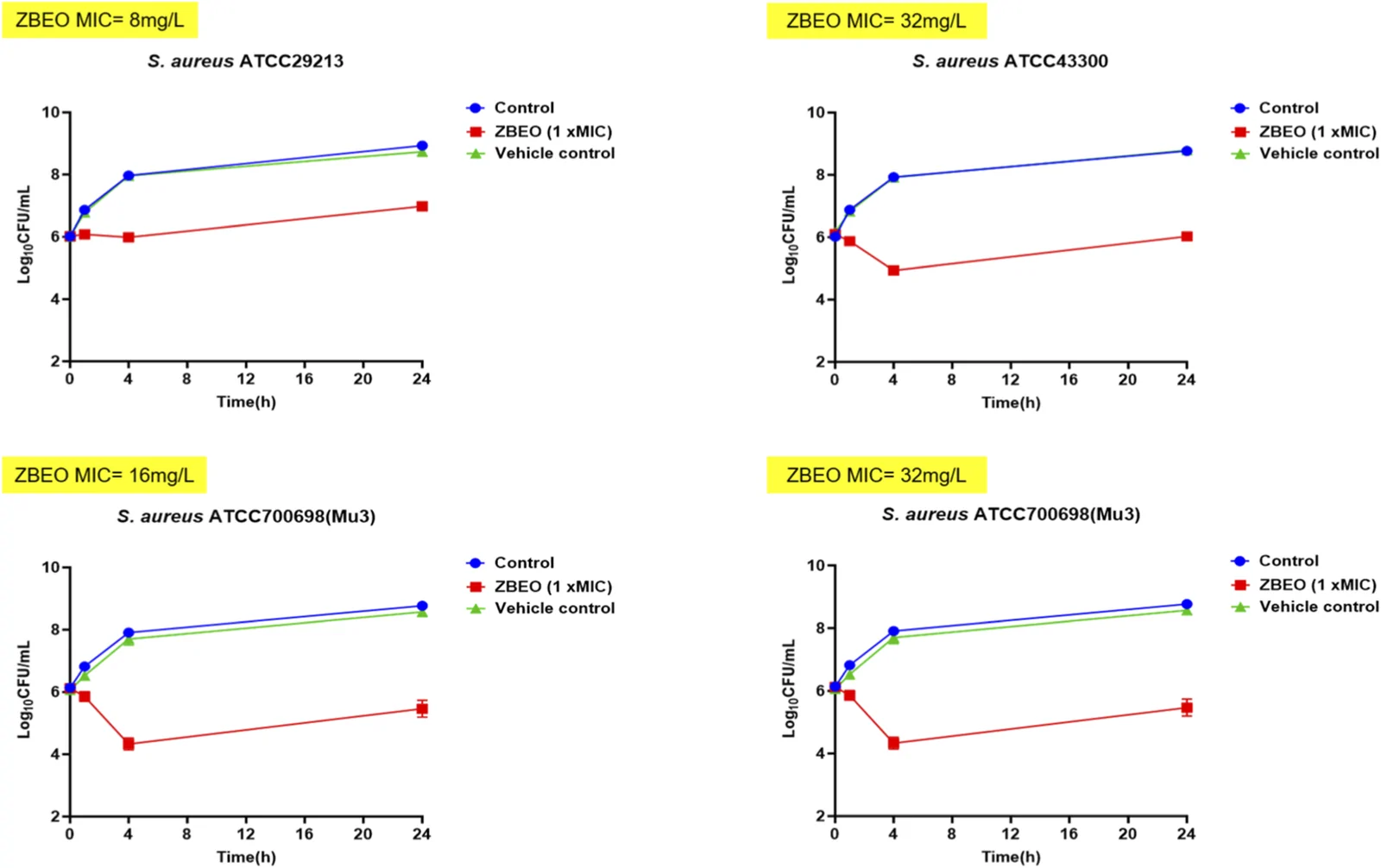
Time-kill kinetics of *Zanthoxylum beecheyanum* essential oil (ZBEO) monotherapy against *Staphylococcus aureus* ATCC 29213, ATCC 43300, JKD6009, and ATCC 700698 (Mu3). ZBEO was tested at 1xMIC for each strain, with corresponding MIC values indicated within individual panels. Untreated bacterial cultures served as the control, while vehicle controls contained 0.05% DMSO. Data are presented as means of three independent cultures. Vertical bars represent standard deviations; error bars were too small to be visible in the graphs.

The bactericidal activity of ZBEO was further evaluated using time-kill assays at 1xMIC against representative *S. aureus* strains, including MSSA, MRSA, hVISA, and clinical isolates. Overall, ZBEO treatment reduced viable bacterial counts relative to untreated controls in all tested strains (Figure 1).

Against *S. aureus* ATCC 29213, ZBEO reduced bacterial counts by approximately 1.9log_10_ CFU/mL relative to the untreated control at 4 h, with this level of inhibition maintained through 24 h. In contrast, greater antibacterial activity was observed against the MRSA strain ATCC 43300, reducing bacterial counts by approximately 2.9 and 2.7 log_10_ CFU/mL at 4 and 24 h, respectively.

The clinical MRSA isolate JKD6009 demonstrated moderate susceptibility to ZBEO treatment. Maximal inhibition was observed at 4 h, corresponding to an average reduction of approximately 3.0 log_10_ CFU/mL, whereas reduced activity was observed at 24 h, with an average reduction of approximately 1.0 log_10_ CFU/mL.

Among the tested strains, the hVISA strain Mu3 (ATCC 700698) showed the greatest susceptibility to ZBEO monotherapy. ZBEO treatment reduced bacterial counts by approximately 3.5 and 3.3 log_10_ CFU/mL relative to the untreated control at 4 and 24 h, respectively. These findings demonstrate that ZBEO exerts time-dependent antibacterial activity against multiple *S. aureus* phenotypes, including MRSA and hVISA strains.

### Combination activity of ZBEO and vancomycin against *Staphylococcus aureus*

#### Checkerboard analysis and fractional inhibitory concentration index (FICI)

Checkerboard analysis demonstrated FICI values ranging from 0.375 to 2.0 across the tested *S. aureus* strains (Table 2).

**TABLE 2:** Antimicrobial activity and checkerboard interaction analysis of vancomycin (VAN) and *Zanthoxylum beecheyanum* essential oil (ZBEO) against *Staphylococcus aureus* isolates.

Strain	MIC (mg/L)		FICI			Interpretation
	VAN	ZBEO	VAN	ZBEO	VAN/ZBEO	
*S. aureus* ATCC 29213	1	8	0.25	1	0.375	Synergistic
*S. aureus* ATCC 43300	1	32	0.25	8	0.50	Synergistic
*S. aureus* ATCC 700699 (Mu50)	8	64	2	32	0.75	Additive
*S. aureus* ATCC 700698 (Mu3)	2	16	0.5	4	0.50	Synergistic
*S. aureus* JKD6008	8	32	4	8	0.75	Additive
*S. aureus* JKD6009	4	32	1	4	0.375	Synergistic
*S. aureus* 440,092	4	32	4	32	2	Indifferent
*S. aureus* 439,819	2	64	1	16	0.75	Additive
*S. aureus* 439,668	1	64	1	8	1.125	Indifferent
*S. aureus* 440,090	1	128	0.5	16	0.625	Additive
*S. aureus* 20,691	4	64	1	16	0.50	Synergistic
*S. aureus* 17,107	0.5	64	0.5	64	2	Indifferent
*S. aureus* 17,084	8	128	2	16	0.375	Synergistic
*S. aureus* 17,120	2	64	2	32	1.5	Indifferent
*S. aureus* 21,471	0.5	64	0.25	16	0.75	Additive

FICI = FIC index = (MIC vancomycin in combination with ZBEO/MIC vancomycin monotherapy) + (MIC ZBEO in combination with vancomycin/MIC ZBEO monotherapy); synergy FIC < 0.5; additivity FIC = 0.5–1.0; indifference FIC = 1–4; antagonism FIC ≥ 4 (not observed).

Synergistic interactions (FICI ≤0.5) were observed against several strains, including *S. aureus* ATCC 29213, ATCC 43300, JKD6009, Mu3 (ATCC 700698), and clinical isolates 20,691 and 17,084. The lowest FICI values (0.375) were observed for ATCC 29213, JKD6009, and isolate 17,084, indicating the strong synergistic interaction between ZBEO and vancomycin.

Additive (FICI >0.5–1.0) or indifferent (FICI >1.0–4.0) effects were observed for Mu50 (ATCC 700699) and several clinical isolates, whereas antagonism was not observed. Higher FICI values were identified for isolates 439,668, 440,092, 17,107, and 17,120, indicating limited activity between ZBEO and vancomycin in these strains.

These findings demonstrated that ZBEO could enhance vancomycin activity against multiple *S. aureus* phenotypes, including MRSA and hVISA associated strains (Table 2).

#### Time-kill analysis of ZBEO and vancomycin combination therapy

Representative strains with the lowest FICI values were selected for confirmatory combination time-kill analysis. Vancomycin was tested at 1x MIC for each strain to mimic clinically relevant bactericidal exposure conditions and to allow assessment of potential regrowth kinetics during prolonged incubation. A fixed ZBEO concentration of 32 mg/L was selected to evaluate its ability to potentiate vancomycin activity across strains with differing intrinsic susceptibilities to ZBEO.

**FIGURE 2:**
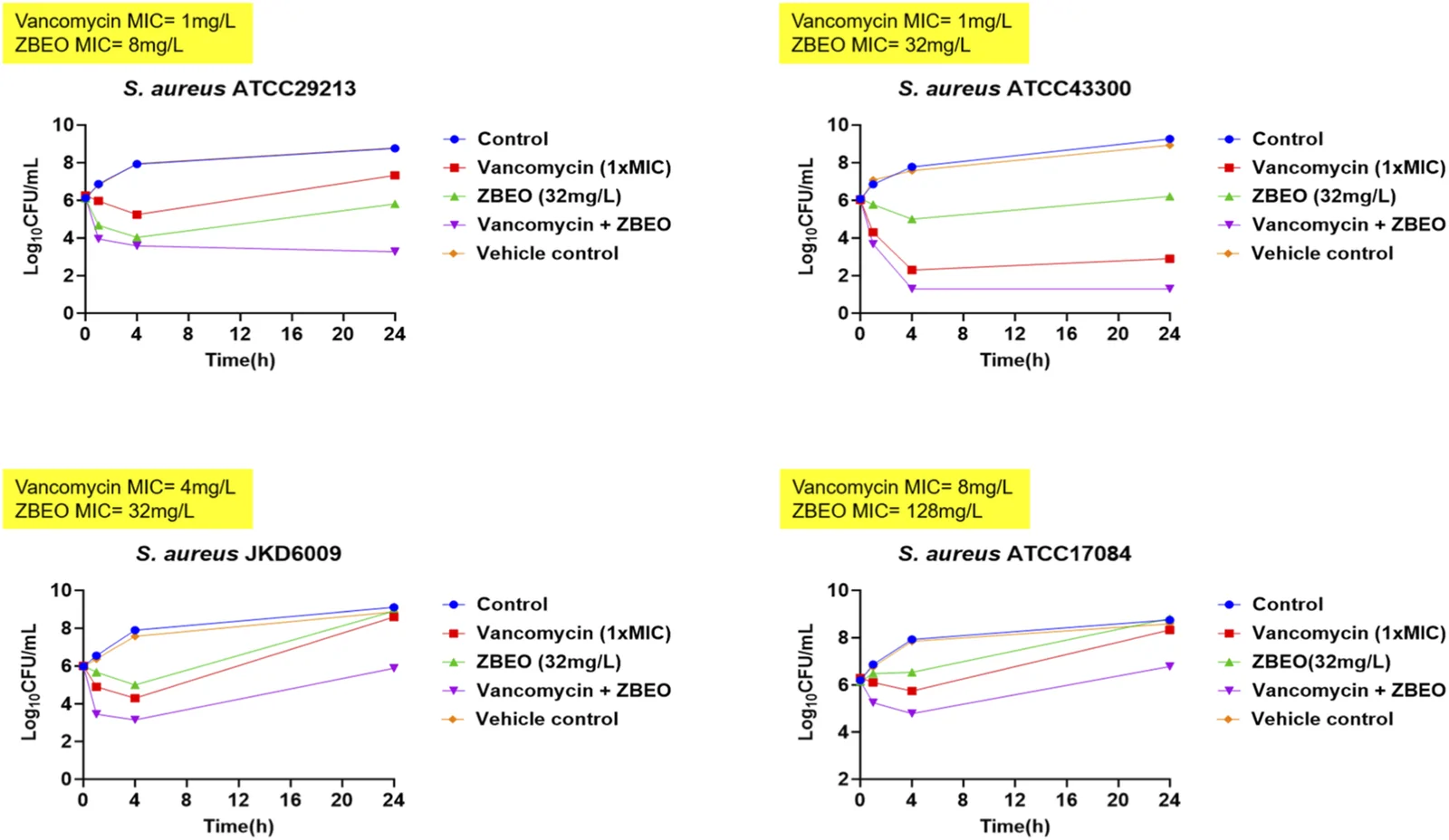
Time-kill kinetics of vancomycin in combination with *Zanthoxylum beecheyanum* essential oil (ZBEO) against *Staphylococcus aureus* ATCC 29213, ATCC 43300, JKD6009, and ATCC 17084. Vancomycin was tested at 1xMIC for each strain in combination with ZBEO (32 mg/L). The corresponding vancomycin MIC values are indicated within the individual panels. Untreated bacterial cultures served as the control, while vehicle controls contained 0.05% DMSO. Data are presented as the mean of three independent biological replicates. Vertical bars represent standard deviations; error bars were too small to be visible in the graphs.

Combination treatment of vancomycin and ZBEO resulted in greater reductions in viable bacterial counts than the untreated controls across all tested strains (Figure 2). The extent of the antibacterial activity differed between strains.

For *S. aureus* ATCC 29213, combination therapy produced substantial bacterial suppression throughout the assay period, with reductions of approximately 2.9, 4.0 and 5.1 log_10_ CFU/mL at 1, 4 and 24 h, respectively, compared to the untreated control. Combination treatment consistently outperformed vancomycin monotherapy at all assessed time points.

Against the MRSA strain *S. aureus* ATCC 43300, combination treatment with vancomycin and ZBEO produced greater and more sustained reductions in bacterial counts than vancomycin monotherapy. Relative to the untreated control, the combination reduced bacterial counts by approximately 3.1, 6.4, and 7.9 log_10_ CFU/mL at 1, 4 and 24 h, respectively, compared with 2.5, 5.4, and 6.3 log_10_ CFU/mL for vancomycin alone.

Similarly, the combination exhibited greater antibacterial activity against *S. aureus* JKD6009, reducing bacterial counts by approximately 3.1, 4.7, and 3.2 log_10_ CFU/mL at 1, 4 and 24 h, respectively, relative to the untreated control. Although vancomycin monotherapy initially reduced bacterial counts during the early time points, substantial regrowth was observed at 24 h, with bacterial counts approaching those of the untreated control. The addition of ZBEO largely prevented bacterial regrowth, resulting in sustained antibacterial activity at 24 h.

In contrast, clinical isolate 17,084 showed the weakest response to the vancomycin-ZBEO combination among the strains tested. Nevertheless, combination treatment reduced bacterial counts by approximately 1.6, 3.1 and 1.9 log_10_ CFU/mL relative to the untreated control at 1, 4 and 24 h, respectively. Although vancomycin monotherapy produced an initial reduction of approximately 2 log_10_ CFU/mL at 4 h, substantial regrowth was observed at 24 h, with bacterial levels approaching those of the untreated control.

Based on the predefined definition of synergy criterion (≥2 log_10_ CFU/mL reduction relative to the most active single agent), synergy was observed for *S. aureus* JKD6009 at 1 and 24 h, ATCC 29213 at 24 h, and clinical isolate 17,084 at 24 h. Although the vancomycin-ZBEO combination enhanced antibacterial activity against ATCC 43300, it did not meet the predefined criterion for synergy.

Overall, these findings demonstrate that ZBEO potentiated vancomycin activity against multiple *S. aureus* strains, with the magnitude of the interaction varying between strains and time points.

#### Antibiofilm activity of ZBEO and vancomycin combination therapy

**FIGURE 3:**
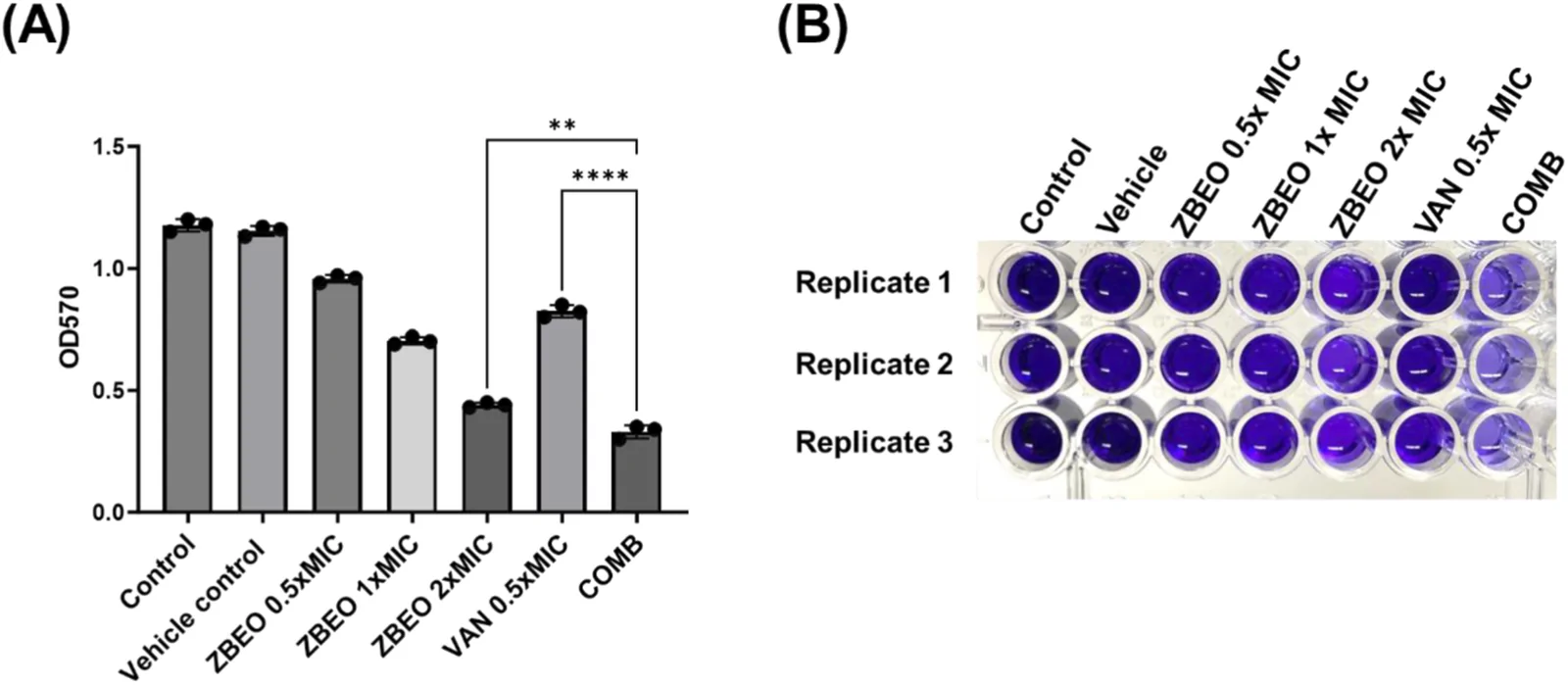
**(A)** Biofilm biomass quantified by crystal violet staining and OD570 measurement following 24 h incubation. **(B)** Representative crystal violet-stained microplate showing three technical replicates for each treatment condition.

The antibiofilm activity of ZBEO alone and in combination with vancomycin was evaluated against *S. aureus* ATCC 43300 using a crystal violet based assay (Figure 3). ZBEO inhibited biofilm formation in a concentration-dependent manner, with approximately 18.9%, 40.6% and 62.7% inhibition observed at 0.5x, 1x and 2xMIC, respectively, relative to the untreated control.

Vancomycin monotherapy at 0.5xMIC reduced biofilm mass by approximately 30.5% relative to the untreated control. In contrast, the combination of ZBEO (2xMIC) and vancomycin (0.5xMIC) produced the greatest reduction in biofilm biomass (72.0%). The combination was significantly more effective than either vancomycin (0.5xMIC) alone (*p* < 0.0001) or ZBEO (2xMIC) alone (*p* = 0.0017) (Figure 3).

The 0.05% dimethyl sulfoxide (DMSO) vehicle control had minimal effect on biofilm formation, with OD570 values comparable to those of the untreated control. These findings demonstrate that ZBEO possesses intrinsic antibiofilm activity and further enhanced the antibiofilm effect of vancomycin against *S. aureus* ATCC 43300 (Figure 3).

### Safety evaluation of ZBEO

#### Hemolytic activity against human erythrocytes

The hemolytic activity of ZBEO against human erythrocytes was evaluated (Table 3) (Demchenko et al., 2021). ZBEO demonstrated low hemolytic activity across the tested concentration range, with HC_10_ ​values exceeding 64 mg/L in all independent experiments. Furthermore, 50% hemolysis was not reached at the highest tested concentration (256 mg/L), with HC_50_ values exceeding 256 mg/L across all replicates.

**TABLE 3:** Hemolytic activity and cytotoxicity of *Zanthoxylum beecheyanum* essential oil (ZBEO).

Assay	Compound	Endpoint	Concentration (mg/L)
Hemolysis (human erythrocytes)	ZBEO	HC_10_	>64
		HC_50_	>256
	Melittin	HC_10_	5.76–6.21
		HC_50_	11.64–13.52
Cytotoxicity (HEK-293, ATCC CRL-1573)	ZBEO	CC_50_	>128
	Tamoxifen	CC_50_	26.06; 46.13

HC_10_ and HC_50_ represent the concentrations corresponding to 10% and 50% hemolysis, respectively. CC_50_ represents the concentration corresponding to a 50% reduction in HEK-293 cell viability. Melittin and tamoxifen were used as the positive controls for the hemolysis and cytotoxicity assays, respectively. Hemolysis assays were performed using human erythrocytes from three independent donors. Cytotoxicity assays were performed using HEK-293 cells (ATCC CRL-1573) in two independent experiments, with each concentration tested in ≥3 technical replicate wells; technical replicates were averaged prior to four parameter logistic curve fitting. Values preceded by “>” indicate that the respective endpoint was not reached at the highest concentration tested (256 mg/L for hemolysis and 128 mg/L for cytotoxicity).

In contrast, the positive control melittin demonstrated substantially greater hemolytic activity, with HC_10_ and HC_50_ values observed at markedly lower concentrations. These findings indicate that ZBEO possesses comparatively low hemolytic activity at concentrations associated with antibacterial effects against susceptible and intermediate strains.

### Cytotoxicity against HEK-293 cells

The cytotoxicity of ZBEO against HEK-293 human embryonic kidney cells was evaluated using a resazurin based viability assay (Table 3) (Demchenko et al., 2021). ZBEO demonstrated limited cytotoxicity toward HEK-293 cells, with CC_50_ values exceeding 128 mg/L in both independent experiments. In comparison, the positive control tamoxifen produced substantially lower CC_50_ values.

## Discussion

The present study demonstrates that the essential oil derived from *Z*. *beecheyanum* exhibits antibacterial activity against a diverse panel of clinically relevant *S. aureus* isolates, including MRSA, hVISA, VISA, and clinical strains with varying susceptibility profiles. Beyond its intrinsic antibacterial activity, ZBEO enhanced the activity of vancomycin, reduced bacterial regrowth during prolonged exposure, and inhibited biofilm formation. These findings support the continued investigation of plant derived secondary metabolites as adjunctive approaches for improving the activity of existing antibiotics against difficult-to-treat *S. aureus* infections (Costerton et al., 1999; Otto, 2018; Cong et al., 2020; Worthington and Melander, 2013). Although antibacterial activity was observed across all isolates, susceptibility varied between strains. Mu50 (VISA) and several clinical isolates exhibited higher MIC values than ATCC 29213 and Mu3 (hVISA), consistent with the reduced susceptibility commonly associated with VISA strains (Howden et al., 2010; Hiramatsu et al., 2014; Howden et al., 2006). However, similarly elevated MIC values among several non-VISA clinical isolates suggest that susceptibility to ZBEO is not determined solely by the VISA phenotype but is likely influenced by additional strain specific factors. This variability is not unexpected given the genetic and phenotypic diversity among clinical *S. aureus* isolates (Tong et al., 2015; Howden et al., 2010).

GC-MS analysis showed that ZBEO was dominated by D-limonene and methyl cinnamate, accompanied by lower abundance terpenoid metabolites. Rather than attributing the observed antibacterial activity to a single compound, the results are more consistent with the collective activity of multiple phytochemicals acting through complementary mechanisms. Essential oils are increasingly recognized as complex mixtures in which additive or synergistic interactions between metabolites contribute substantially to biological activity (Cushnie and Lamb, 2005; Afonso et al., 2023; Silva et al., 2016; Okagu et al., 2021b; Wen et al., 2024; Okagu et al., 2021a). Both D-limonene and methyl cinnamate have previously been associated with antibacterial and antibiofilm properties, while other terpenoids present within the oil may further contribute to membrane perturbation and bacterial stress responses (Okagu et al., 2021a; Sun, 2007; Wen et al., 2024).

One of the principal findings of this study was the ability of ZBEO to enhance vancomycin activity against multiple *S. aureus* strains. Checkerboard analyses demonstrated synergistic effects in several isolates, which were supported by time-kill experiments showing enhanced bacterial suppression and reduced regrowth compared with vancomycin monotherapy. Regrowth following glycopeptide exposure has been associated with adaptive survival responses, heterogeneous resistance, and persistence related phenotypes that contribute to therapeutic failure (Howden et al., 2010; Cong et al., 2020; Balaban et al., 2019; Hussein et al., 2025b). The improved bacterial suppression observed following the addition of ZBEO therefore suggests that the essential oil interferes with bacterial processes that facilitate survival during prolonged antibiotic exposure. Although the precise mechanism remains to be determined, phytochemicals within ZBEO may increase bacterial susceptibility through membrane perturbation, altered cell envelope permeability, oxidative stress induction, or disruption of stress response pathways, mechanisms that have been described for several plant derived antimicrobial compounds (Hussein et al., 2025a; Cushnie and Lamb, 2005; Afonso et al., 2023; Silva et al., 2016).

Previous studies have shown that plant derived extracts and essential oils can enhance the activity of conventional antibiotics against multidrug-resistant *S. aureus*. Although antimicrobial activity has been reported for several *Zanthoxylum* species, few studies have investigated their use in combination with vancomycin against clinically relevant MRSA, hVISA, and VISA strains. Our findings therefore expand the current knowledge by showing that *Z. beecheyanum* essential oil enhances vancomycin activity while also inhibiting biofilm formation in *S. aureus* strains.

The antibiofilm activity observed in the present study further supports the therapeutic potential of ZBEO. Biofilm formation is a major contributor to chronic and device associated *S. aureus* infections by limiting antibiotic penetration and promoting bacterial persistence within protected multicellular communities (Cong et al., 2020; Costerton et al., 1999). ZBEO alone inhibited biofilm formation in a concentration-dependent manner, while combination treatment with vancomycin produced the greatest reduction in biofilm biomass. Although the underlying mechanism was not investigated, these findings suggest that ZBEO may interfere with early biofilm establishment or enhance the activity of vancomycin against biofilm associated cells. These effects may be particularly relevant for persistent staphylococcal infections in which biofilm formation contributes to poor clinical outcomes.

Preliminary safety evaluation demonstrated limited hemolytic activity and low cytotoxicity toward HEK-293 cells at concentrations relevant to the antibacterial activity observed against susceptible and intermediate isolates. Although these assays provide only an initial indication of safety, they suggest that ZBEO does not exhibit marked nonspecific toxicity at biologically active concentrations. Given that many membrane active natural products display limited selectivity because of concurrent toxicity toward mammalian cells, these findings provide encouraging preliminary support for further preclinical evaluation.

Several limitations should be acknowledged. The study evaluated a chemically characterized essential oil rather than purified individual constituents; therefore, the compounds responsible for the observed antibacterial and vancomycin potentiating activities remain to be identified. Future studies should employ bioassay guided fractionation together with preparative chromatography and structural characterization using techniques such as NMR, LC-MS/MS, and HRMS to identify the bioactive metabolites responsible for the observed antibacterial activity. In addition, further work is required to determine the physicochemical properties, formulation stability, and pharmacokinetic profile of ZBEO before its therapeutic potential can be fully evaluated. Furthermore, mechanistic studies investigating membrane integrity, oxidative stress, quorum sensing, or transcriptional responses will be important to define the mechanisms underlying the antibacterial and vancomycin-potentiating effects of ZBEO. Overall, the present findings identify *Z. beecheyanum* essential oil as a promising source of adjunctive antibacterial agents that warrant further mechanistic investigation and preclinical evaluation against resistant *S. aureus* infections.

## Materials and methods

### Chemicals and reagents

Vancomycin hydrochloride, DMSO, crystal violet, resazurin sodium salt, melittin, phosphate buffered saline (PBS), Cation Adjusted Mueller Hinton broth (CAMHB), Dulbecco’s modified Eagle medium (DMEM), fetal bovine serum (FBS), and other analytical grade reagents were purchased from Sigma-Aldrich unless otherwise stated. Tryptic soy agar and nutrient agar were obtained from Oxoid. Human whole blood used for hemolysis assays was purchased from Innovative Research (USA).

### Plant material collection

Fresh leaves of *Zanthoxylum beecheyanum* K. Koch were collected from the medicinal plant garden of the College of Pharmacy, Al-Mustansiriya University, Baghdad, Iraq. The plant material was taxonomically authenticated by qualified botanists at the National Herbarium of Iraq, where a voucher specimen (No. C-1187) was deposited. The collected leaves were visually inspected to remove damaged material and were processed immediately after collection for essential oil extraction.

### Essential oil extraction

Fresh leaves of *Z. beecheyanum* (100.10 g) were subjected to hydrodistillation using a Clevenger type apparatus (Jeulin, Évreux Cedex, France) for 3 h. Distillation was performed using distilled water at approximately 70 °C. The essential oil was collected, separated from the aqueous phase, and dried over anhydrous sodium sulfate to remove residual moisture (Fan et al., 2018; Kusuma and Mahfud, 2017). The oil was then transferred to sealed amber glass vials and stored at 4 °C until GC-MS analysis and antimicrobial testing.

For biological assays, 50 mg of essential oil was dissolved in 1 mL of DMSO to prepare a stock solution (50 mg/mL) (Kusuma and Mahfud, 2017; Fan et al., 2018). Working concentrations were prepared in CAMHB. The final DMSO concentration did not exceed 0.05% (v/v).

### Phytochemical characterization of the essential oil

The volatile composition of the hydrodistilled *Z. beecheyanum* essential oil was characterized by gas chromatography-mass spectrometry (GC-MS) using an Agilent 7890B gas chromatograph coupled to an Agilent 5977A mass selective detector (Agilent Technologies, Santa Clara, CA, USA). Separation was performed on an Agilent HP-5ms Ultra Inert capillary column (30 m × 0.25 mm i. d., 0.25 μm film thickness). Helium (99.99%) was used as the carrier gas at a constant flow rate of 1.0 mL/min with an inlet pressure of 7.07 psi. Samples (1 μL) were injected in pulsed splitless mode. The injector, GC inlet line, and auxiliary heater temperatures were maintained at 290 °C, 250 °C, and 300 °C, respectively. The oven temperature program was as follows: an initial temperature of 60 °C was held for 3 min, increased to 180 °C at 7 °C/min, then increased to 280 °C at 8 °C/min and held for 5 min, giving a total run time of 40 min. Mass spectra were acquired over an m/z range of 35–650. Volatile metabolites were tentatively identified by comparison of their mass spectra with the National Institute of Standards and Technology (NIST) mass spectral library together with retention times. Relative abundances were estimated from chromatographic peak area percentages. The identified volatile metabolites are listed in Supplementary Table S1. Retention indices and authentic reference standards were not used in the present study.

### Bacterial strains and culture conditions

Antibacterial experiments were performed using a panel of clinically relevant *S. aureus* strains, including methicillin-susceptible *S. aureus* (MSSA), methicillin-resistant *S. aureus* (MRSA), heterogeneous vancomycin-intermediate *S. aureus* (hVISA), vancomycin-intermediate *S. aureus* (VISA), and clinical isolates.

Bacterial stocks were maintained at −80 °C in cryovials containing tryptic soy broth supplemented with 20% glycerol. Prior to experimentation, strains were subcultured on nutrient agar and propagated in CAMHB.

### Determination of MICs and FICI

MICs of vancomycin and ZBEO were determined using the broth microdilution method according to Clinical and Laboratory Standards Institute (CLSI) guidelines (Weinstein, 2018). Two-fold serial dilutions of both vancomycin and ZBEO ranging from 0.125 to 256 mg/L were prepared. ZBEO was dissolved in DMSO and diluted in CAMHB. Vancomycin was freshly prepared in sterile Milli-Q water. Bacterial suspensions were adjusted to approximately 0.5 McFarland standard. For each well, 100 µL of bacterial suspension was mixed with 100 µL of serially diluted antimicrobial solutions, resulting in a final volume of 200 µL per well. Plates were incubated at 37 °C for 18–20 h. MIC was defined as the lowest concentration showing complete inhibition of visible bacterial growth (Weinstein, 2018).

Vancomycin MICs were interpreted according to CLSI breakpoints for *S. aureus*: susceptible ≤2 mg/L, intermediate 4–8 mg/L, and resistant ≥16 mg/L (Institute, 2024).

Checkerboard assays were performed to evaluate the synergy between vancomycin and ZBEO. The FICI was calculated as follows:

$$FICI=\left(\frac{\text{MIC}\;\text{of}\;\text{drug}\;A\;\text{in}\;\text{combination}}{\text{MIC}\;\text{of}\;\text{drug}\;A\;\text{alone}}\right)+\left(\frac{\text{MIC}\;\text{of}\;\text{drug}\;B\;\text{in}\;\text{combination}}{\text{MIC}\;\text{of}\;\text{drug}\;B\;\text{alone}}\right)$$

FICI values were interpreted as follows: synergism, FICI ≤0.5; additivity, FICI >0.5–1.0; indifference, FICI >1–4; and antagonism, FICI ≥4. All checkerboard assays were performed in three independent biological replicates, and the same FICI classification was obtained across all biological replicates for each strain.

### Static time-kill assays

Static time-kill assays were performed to evaluate the antibacterial activity of ZBEO alone and in combination with vancomycin against selected *S*. *aureus* strains. Overnight bacterial cultures were prepared by inoculating single colonies into 10 mL of CAMHB and incubating at 37 °C with shaking at 150 rpm for 16–18 h. Cultures were subsequently diluted into fresh CAMHB and incubated for an additional 2 h to obtain early logarithmic-phase growth.

For each assay, early log-phase cultures were diluted 1:100 into fresh CAMHB in sterile glass culture tubes to achieve a final inoculum of approximately 1 x 10^6^ CFU/mL. ZBEO monotherapy was evaluated at 1x MIC for each strain. Combination experiments were conducted with vancomycin at 1xMIC together with a fixed ZBEO concentration of 32 mg/L. A vehicle control containing 0.05% DMSO, corresponding to the final solvent concentration used for ZBEO preparation, was included to assess potential solvent effects. Untreated control groups received neither antimicrobial agent nor DMSO.

All cultures were incubated at 37 °C with shaking at 150 rpm. Samples were collected at 0, 1, 4, and 24 h following treatment, serially diluted in sterile saline, and plated onto nutrient agar for viable colony enumeration. Colony forming units were enumerated after 24 h incubation at 37 °C. Time-kill curves were generated by plotting log_10_ CFU/mL against time (h). Bactericidal activity was defined as a reduction of ≥3-log_10_ CFU/mL relative to the initial inoculum at any time point. Synergy was defined as a ≥2-log_10_ CFU/mL reduction produced by the combination compared with the most active single agent (Standards and Barry, 1999).

### Crystal violet biofilm inhibition assay

Biofilm inhibition activity was evaluated against *S. aureus* ATCC 43300 using a modified crystal violet microtiter plate assay as previously described (O'Toole, 2011). Serial dilutions of ZBEO were prepared in CAMHB and transferred into sterile flat bottomed 96-well microtiter plates (Corning, Cat. no. 3370). ZBEO was tested at 0.5x, 1x, and 2xMIC either alone or in combination with vancomycin at 0.5xMIC.

A 0.5 McFarland bacterial suspension was prepared in sterile 0.9% NaCl and subsequently diluted 1:100 in CAMHB to obtain a final inoculum of approximately 1 × 10^6^ CFU/mL. Aliquots (100 µL) of the bacterial suspension were added to wells containing 100 µL of antimicrobial solution, resulting in a final volume of 200 µL per well. Vehicle control wells containing 0.05% DMSO and untreated growth controls were included.

Plates were placed in sealed humidified containers containing damp paper towels to minimize evaporation and incubated statically at 37 °C for 24 h. Following incubation, planktonic cells were removed and wells were washed three times with phosphate-buffered saline (PBS, pH 7.4). Biofilms were stained with 0.1% crystal violet for 10 min at room temperature. Excess stain was removed by washing with PBS, and retained crystal violet was solubilized using 30% acetic acid.

The turbidity of each well was measured at an optical density of 570 nm using a Tecan M200 microplate reader (Tecan Group Ltd., Männedorf, Switzerland). Biofilm inhibition percentages were calculated relative to untreated controls using the following equation:

$$\%\text{ inhibition}=\frac{OD_{\text{control}}-OD_{\text{treated}}}{OD_{\text{control}}}\times 100$$

### Cytotoxicity assay

Human embryonic kidney cells (HEK-293; ATCC CRL-1573) were maintained in Dulbecco’s modified Eagle medium (DMEM; Gibco, Thermo Fisher Scientific) supplemented with 10% fetal bovine serum (FBS). Cell numbers were determined using a Neubauer hemocytometer prior to seeding into 384-well tissue culture-treated plates (Corning) containing pre-dispensed test compounds. Cells were seeded at a density of 5,000 cells per well in a final volume of 50 µL and incubated at 37 °C in 5% CO_2_ for 20 h.

Cell viability was assessed using a resazurin reduction assay as previously described with minor modifications (Blaskovich et al., 2021; O'Brien et al., 2000). Briefly, 5 µL of resazurin solution (25 μg/mL; Sigma-Aldrich) was added to each well to achieve a final concentration of 2.3 μg/mL, followed by incubation for 3 h at 37 °C. Fluorescence was measured using a Tecan Infinite M1000 Pro plate reader with excitation/emission wavelengths of 560/10 nm and 590/10 nm, respectively. Cytotoxicity values (CC_50_) were calculated using four parameter logistic regression analysis of percentage inhibition against log_10_-transformed concentrations. Each concentration was tested in at least three technical replicates, and experiments were performed independently on two separate occasions.

### Hemolysis assay

Hemolytic activity was evaluated using a human erythrocyte hemolysis assay with minor modifications (Blaskovich et al., 2021). Human whole blood from three independent donors was purchased from Innovative Research Inc. and processed according to the manufacturer’s recommendations. Erythrocytes were washed three times with sterile 0.9% NaCl and resuspended in saline to a final density of 0.5 x 10^8^ cells/mL, determined using a Neubauer hemocytometer.

Aliquots of the washed erythrocyte suspension were added to 384-well polystyrene plates (Corning) containing pre-dispensed test compounds to a final volume of 50 µL per well. Plates were gently shaken for 10 min and incubated at 37 °C for 1 h. Following incubation, plates were centrifuged at 1,000 x g for 10 min, and 25 µL of supernatant was transferred to fresh 384-well plates for analysis.

Hemoglobin release was quantified by measuring absorbance at 405 nm using a Tecan Infinite M1000 Pro plate reader. Percentage hemolysis was calculated relative to vehicle-treated controls (0% hemolysis) and melittin treated controls representing complete lysis. HC_10_ and HC_50_ values were determined using four parameter logistic regression analysis of percentage hemolysis against log_10_-transformed concentrations. Assays were performed using blood obtained from three independent donors, with each concentration tested in at least three technical replicates.

### Statistical analysis

All experiments were performed using at least three independent replicates unless otherwise stated. Data are presented as mean ± standard deviation (SD). Statistical analyses were performed using GraphPad Prism version 10 (GraphPad Software, San Diego, CA, USA).

Biofilm inhibition data were analyzed using ordinary one-way analysis of variance (ANOVA) followed by Tukey’s multiple-comparisons test. Adjusted *p* values <0.05 were considered statistically significant. For cytotoxicity and hemolysis assays, CC_50_, HC_10_, and HC_50_ values were determined using four parameter nonlinear regression using GraphPad Prism 10.

## Data Availability

The original contributions presented in the study are included in the article/Supplementary Material, further inquiries can be directed to the corresponding authors.
