# Supplementary material for: Zanthoxylum beecheyanum enhances vancomycin activity and inhibits biofilm formation in resistant staphylococcus aureus
Source: Front Pharmacol. 2026 Aug 19;17:1919407. doi: 10.3389/fphar.2026.1919407 (PMC13534080; doi:10.3389/fphar.2026.1919407)
Supplement: Supplementary file 1 [file DataSheet1.docx]

**Supplementary Information: *Zanthoxylum beecheyanum* Enhances Vancomycin Activity and Inhibits Biofilm Formation in Resistant *Staphylococcus aureus***

Arshed Idrees,^1^ Ibrahim Salih Abbas,^1^ Jian Li,^2^ Maytham Hussein,^3^* Tony Velkov^3^*

**Affiliations:** ^1^Department of Pharmacognosy, College of Pharmacy, Mustansiriyah University, Baghdad, Iraq; ^2^Monash Biomedicine Discovery Institute, Department of Microbiology, Monash University, Clayton, Victoria, 3800, Australia; ^3^Monash Biomedicine Discovery Institute, Department of Pharmacology, Monash University, Clayton, Victoria, 3800, Australia;

*Corresponding author: tony.velkov@monash.edu OR maytham.hussein.old@monash.edu

**Short title:** Adjunctive Plant Derived Therapy against Resistant *S. aureus*

**Keywords:** *Staphylococcus aureus*; MRSA; vancomycin synergy; biofilm inhibition; *Zanthoxylum beecheyanum*; adjunctive therapy

**Supplementary Figure S1.** Total ion chromatogram (TIC) obtained by GC-MS analysis of the essential oil of *Zanthoxylum beecheyanum* K. Koch.

**Supplementary Table S1.** Major volatile constituents identified in *Zanthoxylum beecheyanum* essential oil by GC-MS.

| Peak | RT (min) | Compound | Area (%) |
| --- | --- | --- | --- |
| 1 | 9.091 | β-Pinene | 6.22 |
| 2 | 10.326 | D-Limonene | 50.85 |
| 3 | 11.018 | Cyclohexene, 1-methyl-4-(1-methylethylidene)- | 0.72 |
| 4 | 11.609 | Octanoic acid, methyl ester | 0.58 |
| 5 | 15.024 | α-Terpinyl acetate | 0.61 |
| 6 | 16.001 | Methyl cinnamate [(E)-3-phenyl-2-propenoic acid methyl ester] | 38.30 |
| 7 | 16.137 | Caryophyllene | 1.16 |
| 8 | 16.565 | 1,4,7-Cycloundecatriene, 1,5,9,9-tetramethyl-, Z,Z,Z- | 1.56 |
